# Profiling and Functional Analysis of microRNA Deregulation in Cancer-Associated Fibroblasts in Oral Squamous Cell Carcinoma Depicts an Anti-Invasive Role of microRNA-204 via Regulation of Their Motility

**DOI:** 10.3390/ijms222111960

**Published:** 2021-11-04

**Authors:** Saroj Rajthala, Anjie Min, Himalaya Parajuli, Kala Chand Debnath, Borghild Ljøkjel, Kristin Marie Hoven, Arild Kvalheim, Stein Lybak, Evelyn Neppelberg, Olav Karsten Vintermyr, Anne Christine Johannessen, Dipak Sapkota, Daniela Elena Costea

**Affiliations:** 1 Gade Laboratory for Pathology, Department of Clinical Medicine, Faculty of Medicine, University of Bergen, 5020 Bergen, Norway; saroj.rajthala@uib.no (S.R.); william0732@csu.edu.cn (A.M.); himalaya.parajuli@uib.no (H.P.); kcdebnath.cdc@gmail.com (K.C.D.); Olav.Karsten.Vintermyr@helse-bergen.no (O.K.V.); anne.johannessen@uib.no (A.C.J.); 2 Centre for Cancer Biomarkers (CCBIO), Faculty of Medicine, University of Bergen, 5020 Bergen, Norway; 3 Department of Oral Maxillofacial Surgery, Xiangya Hospital, Central South University, Changsha 410008, China; 4 Head and Neck Clinic, Haukeland University Hospital, 5021 Bergen, Norway; borghild.ljokjel@helse-bergen.no (B.L.); kristin.marie.hoven@helse-bergen.no (K.M.H.); stein.lybak@helse-bergen.no (S.L.); evelyn.neppelberg@helse-bergen.no (E.N.); 5 Oral Surgery Private Referral Practice “Tannteam”, 5221 Nesttun, Norway; ari-kval@online.no; 6 Department of Oral Surgery, Institute of Clinical Dentistry, University of Bergen, 5020 Bergen, Norway; 7 Department of Pathology, Haukeland University Hospital, 5020 Bergen, Norway; 8 Department of Oral Biology, University of Oslo, 0372 Oslo, Norway; dipak.sapkota@odont.uio.no

**Keywords:** oral squamous cell carcinoma, miR-204, fibroblasts, tumor stroma, ITGA11

## Abstract

*Background:* Knowledge on the role of miR changes in tumor stroma for cancer progression is limited. This study aimed to investigate the role of miR dysregulation in cancer-associated fibroblasts (CAFs) in oral squamous cell carcinoma (OSCC). *Methodology:* CAF and normal oral fibroblasts (NOFs) were isolated from biopsies of OSCC patients and healthy individuals after informed consent and grown in 3D collagen gels. Total RNA was extracted. Global miR expression was profiled using Illumina version 2 panels. The functional impact of altered miR-204 expression in fibroblasts on their phenotype and molecular profile was investigated using mimics and inhibitors of miR-204. Further, the impact of miR-204 expression in fibroblasts on invasion of adjacent OSCC cells was assessed in 3D-organotypic co-cultures. *Results:* Unsupervised hierarchical clustering for global miR expression resulted in separate clusters for CAF and NOF. SAM analysis identified differential expression of twelve miRs between CAF and NOF. Modulation of miR-204 expression did not affect fibroblast cell proliferation, but resulted in changes in the motility phenotype, expression of various motility-related molecules, and invasion of the adjacent OSCC cells. 3′ UTR miR target reporter assay showed ITGA11 to be a direct target of miR-204. *Conclusions:* This study identifies differentially expressed miRs in stromal fibroblasts of OSCC lesions compared with normal oral mucosa and it reveals that one of the significantly downregulated miRs in CAF, miR-204, has a tumor-suppressive function through inhibition of fibroblast migration by modulating the expression of several different molecules in addition to directly targeting ITGA11.

## 1. Introduction

Oral cancer represents a significant proportion of head and neck cancer and is an important cause of morbidity and mortality worldwide [1]. It was recently estimated that an alarming 109% increase in the number of incident lip and oral cavity cancers over a 28 y period has occurred (from around 186,000 in 1990 to 389,800 in 2017) [2]. Oral squamous cell carcinoma (OSCC) that arises from oral epithelium represents 90% of the oral cancers [3]. Although few studies showed an improvement in survival for OSCC during the last years [4], owing to recent advances in treatment of SCC [5,6], many studies have reported that the five-year survival for OSCC remained low during the last decades, and thus it requires a better understanding of its biology for designing even more innovative treatment modalities [7,8].

The discovery of micro-RNAs (miRs) as regulators of gene expression [9,10,11], and evidence of their importance for development and disease [12], has led to a burst of miR studies in cancers, including OSCC. Since then, several miRs have been shown to have either tumor-promoting or tumor-suppressive roles [13]. For example, miR-21, miR-146a, miR-155, and miR-134 have been shown to exhibit oncogenic functions, while miR-7, miR-99a, and miR-218 have been proven to exhibit tumor suppressive functions (reviewed in [14]) in OSCC.

With regards to changes in miR expression in tumors, miRs have been suggested as potential diagnostic and prognostic biomarkers in several cancers. Changes in miR expression in various biological samples such as tumor biopsies, serum/plasma, and saliva have been reported. For example, miR-21 has been reported to be increased in both plasma and tissues in OSCC, and miR-29a has been shown to be decrease in serum of OSCC patients. More than that, miRs have also been regarded as potential targets for therapeutic intervention (reviewed in [14,15,16]).

Several lines of evidence support now that it is not only the intrinsic properties of the epithelial cells that drive carcinogenesis. Rather, the invasion potential of transformed cancer cells is largely influenced by the tumor microenvironment composed of fibroblasts, immune cells, blood and lymph vessels, muscles, fat, nerves, extracellular matrix, and soluble factors [17,18]. Fibroblasts are the most abundant mesenchyme-derived cell type in the stroma responsible for the structural framework of tissues. Cancer-associated fibroblasts (CAFs), which can be derived from several sources, such as neighboring normal fibroblasts, pericytes, endothelial cells, or from mesenchymal stem cells from bone marrow, have been shown to play an important role in supporting tumor initiation and invasion in both in vitro cell culture studies [19,20,21] and in vivo animal studies [19]. In addition, CAF have been associated with lymph node metastasis [22,23] and poor prognosis [22,23,24,25,26].

While most studies focused on miR changes in tumor cells or tumor as a whole, including in the OSCC studies, our knowledge on miR alterations in the tumor microenvironment, and in particular in CAFs, is very sparse. Given the impact of both miRs and CAFs in tumor progression, miR dysregulation in CAFs could be a major factor in determining the behavior of tumor cells. Thus, this study was aimed at investigating miR dysregulation in CAFs in OSCC. Our previous transcriptomic study that compared the same CAF and NOF strains used in this study identified altered expression of integrin alpha 11 (ITGA11) in CAFs [19]. We have also validated this finding on patient material, by detected significantly increased expression of ITGA11 in the stroma of head and neck squamous cell carcinoma compared with normal oral mucosa controls [24]. This further led our investigation towards miR-204, one of the miRs identified by the microarray study presented here to have an altered expression in CAFs and to have a predicted target site at 3′ UTR of ITGA11.

Several studies have shown decreased expression of miR-204 in tumor tissues compared with normal tissues [27,28,29,30], but none investigated specifically its alterations in CAFs. An anti-tumorigenic effect of miR-204 has been demonstrated in cell culture and in animal studies [27,28,29,30,31], using cancer cells, but not CAFs. Lower expression of miR-204 has been observed in oral premalignant lesions [32] and OSCC tumors compared with normal tissues [33,34], and the anti-tumor effect of miR-204 has also been demonstrated in vitro in OSCC cells, but not in OSCC-derived CAFs [35]. In a recent study on a cohort of 169 patients with human papilloma virus (HPV)-negative primary OSCC, we showed that presence of miR-204 in the stroma at the tumor front predicted better overall survival and recurrence free survival [36]. Nevertheless, none of these previous studies investigated the dysregulation of miR-204 in CAFs and its impact on the behavior of OSCC cells. Therefore, this study sought also to explore the functional role of expression of miR-204 in CAFs on OSCC progression.

## 2. Results

### 2.1. miR Array Identifies Twelve miRs with Differential Expression in CAFs versus NOFs

**Figure 1:**
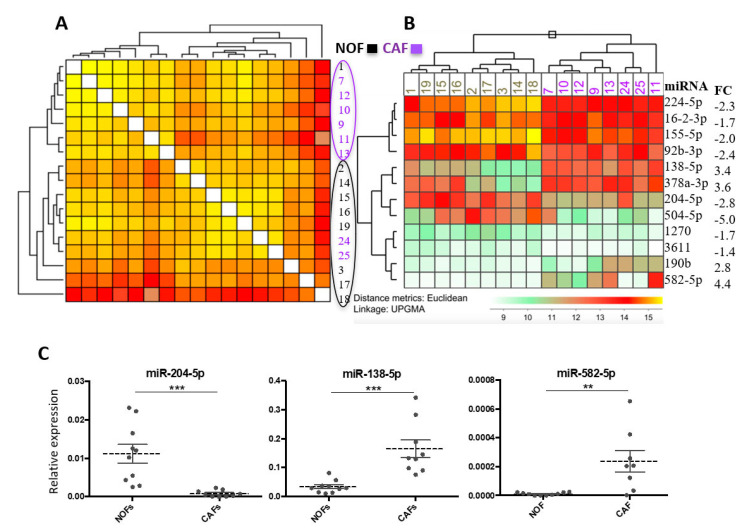
(**A**) Unsupervised hierarchical sample clustering of CAFs and NOFs for miR expression. (**B**) Clustering analysis by fibroblast type for 12 significantly differentially regulated miRs (SAM analysis). Significantly altered miRs (FDR = 0) and corresponding fold changes (FCs) on the right. (**C**) miR validation of microarray results by qRT-PCR (** *p* < 0.005, *** *p* < 0.0005).

Unsupervised hierarchical clustering performed on all the samples (unmatched CAFs and NOFs grown in 3D collagen gels) based upon global miR expression resulted in separate clusters of CAFs (lilac ring) and NOFs (black ring), although with some minor inter-clustering; two CAF strains clustered together with NOFs, and one strain of NOF clustered together with CAFs (Figure 1A). SAM analysis identified twelve significantly (FDR = 0) differential regulated miRs (Figure 1B). Different subgroups for CAFs and NOFs were observed when clustered for the twelve significantly differentially regulated miRs by fibroblast type (Figure 1B). Four of the miRs: miR-138-5p, miR-378a-3p, miR-190b, and miR-582-5p were significantly up-regulated, while miR-224-5p, miR-16-2-3p, miR-155-5p, miR-92b-3p, miR-204-5p, miR-504-5p, miR-1270, and miR-3611 were significantly down-regulated in CAFs compared with NOFs (Figure 1B).

### 2.2. Validation of Altered Expression of miR-204 in Cultured Fibroblasts and Patient Material

**Figure 2:**
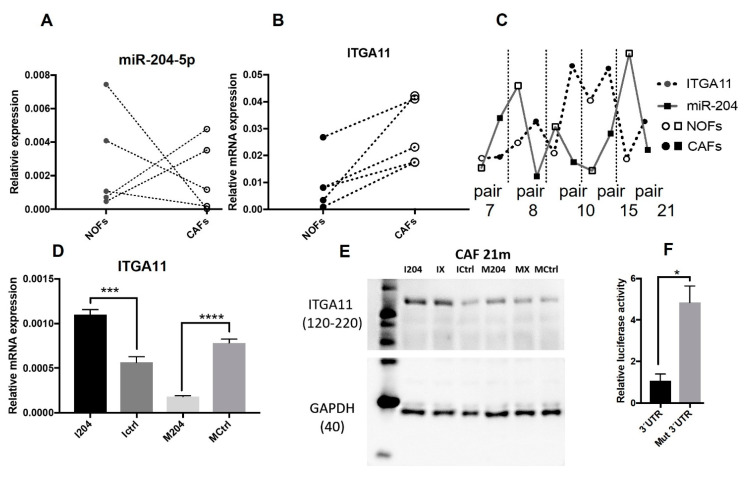
ITGA11 and its regulation by miR-204: relative expression of (**A**) miR-204 and (**B**) ITGA11 in NOFs compared with matched CAFs (connected by dotted lines), and the corresponding (**C**) expression correlation between miR-204 and ITGA11. (**D**) mRNA and (**E**) protein (Western blot image) regulation of ITGA11 by miR204, 48 h post transfection. (**F**) Luciferase activity in CAFs in one with miR-204 target site and another with mutated sequence in 3′ UTR for ITGA11 in miR target reporter assay (* *p* < 0.05, *** *p* < 0.0005, **** *p* < 0.00005, unpaired Student’s *t*-test, *n* = 4).

qRT-PCR validation of some of the dysregulated miRs (miR-204-5p, miR-138-5p, and miR-582-5p) confirmed the results of the miR array analysis (Figure 1C). Unlike down-regulation of miR-204 expression in CAFs compared with unmatched NOFs, the alterations in the expression of miR-204 in CAFs compared with NOFs were more heterogeneous when investigated on matched CAF and NOF pairs from OSCC patients grown in 2D cultures. Three (ID 8m, 10m, and 21m) of the five fibroblast pairs showed reduced expression of miR-204 in CAFs compared with NOFs (Figure 2A), while in two pairs’ (ID 7m and 15m) miR-204 expression in was increased in CAFs versus NOFs.

**Figure 3:**
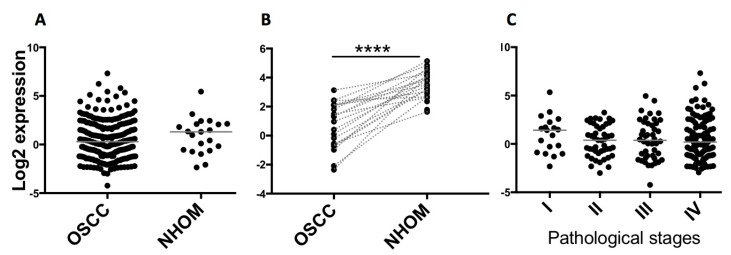
Graphs showing expression of miR-204 in OSCC (TCGA data): (**A**) No significant difference between miR204 expression in OSCC compared with normal tissues (Mann-Whitney test; *n* = 251 OSCC, *n* = 21 NHOM). (**B**) A significant difference was detected between miR-204 expression in matched OSCC-NHOM cases (paired *t*-test; *n* = 20, matched cases are linked by a dotted line, **** *p* < 0.00005). (**C**) No significant difference in miR-204 expression among different OSCC stages (Kruskal-Wallis test showed no significant difference between different stages, although a trend for decreased expression from stage I to more advanced stages could be observed).

When TCGA data on expression of miR-204 in tumor as a whole were analyzed, a statistically significant difference in expression of miR-204 between OSCC lesions and normal human oral mucosa was not observed (Figure 3A). However, statistically significant (*p* < 0.001) down-regulation of miR-204 was observed when OSCC lesions were compared with their matched normal oral mucosa (Figure 3B). Despite statistically insignificant differences among OSCC pathological stages, a trend for decreased expression from stage I to more advanced stages was observed (Figure 3C).

### 2.3. ITGA11 Is a Direct Target of miR-204 in Fibroblasts

The same CAF and NOF strains analyzed here were previously investigated for their transcriptomic differences [19]. mRNA for ITGA11 was identified as one of the core mRNAs upregulated in CAFs versus NOFs. Prediction of miR targets for ITGA11 using TargetScan 7.2 showed conserved pairing of miR-204 seed region in the 3′ UTR length of ITGA11 [33]. qRT PCR showed that ITGA11 transcript was significantly higher in CAFs compared with NOFs in all five fibroblast matched pairs investigated here (Figure 2B). An inverse correlation between expressions of miR-204 and ITGA11 was observed in three out of five matched fibroblast pairs (ID 8m, 10m, and 21m (Figure 2C)).

Furthermore, increased expression of miR-204 in fibroblasts using mimics resulted in decreased expression of ITGA11 both at the mRNA (Figure 2D) and protein (Figure 2E) level. On the contrary, inhibition of miR-204 resulted in increased expression of ITGA11, both at the mRNA and protein level, pointing also towards a direct regulation of ITGA11 by miR-204.

Finally, miR target reporter assay confirmed the direct targeting of ITGA11 by miR-204 in CAFs. Co-transfection of miR-204 mimics and the reporter vector bearing 3′ UTR length of ITGA11 into CAFs resulted in significantly reduced luciferase activity by 77.85%, compared with the control bearing mutation in the miR-204 seed binding motif of 3′ UTR length of ITGA11 (Figure 2F).

### 2.4. miR-204 Modulates the Expression of Several CAF-Related Molecules

**Figure 4:**
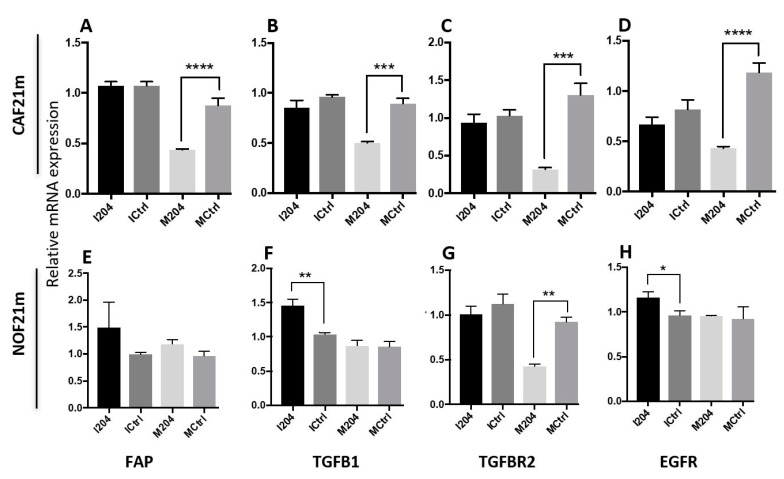
Regulation of CAF-related transcripts by miR-204 ((* *p* < 0.05, ** *p* < 0.005, *** *p* < 0.0005, and **** *p* < 0.0001, unpaired Student’s *t*-test, *n* = 3). (**A**,**E**) FAP expression; (**B**,**F**) TGFB1 expression; (**C**,**G**) TGFB2 expression; and (**D**,**H**) EGFR expression in CAF21m and NOF21m respectively.

Transfection of CAFs with miR-204 mimics induced a significant decrease in mRNA and protein levels of several molecules that are considered to be characteristic of CAF phenotype, such as FAP and TGFβ-related molecules (TGFβ1 and TGFBR2) [37] (Figure 4A–C, Figure S1). Expression levels of mRNA for EGFR were also downregulated, while miR-204 mimics were transfected (Figure 4D). Opposite effects after transfection with miR-204 inhibitors were not observed. In NOFs, a significant effect of miR-204 mimics was observed for TGFBR2 only (Figure 4G), while inhibition of miR-204 led to a significant increase in expression of TGFB1 and EGFR (Figure 4F,H).

### 2.5. miR-204 Decreases Migration and Collagen Contraction Abilities of CAFs

**Figure 5:**
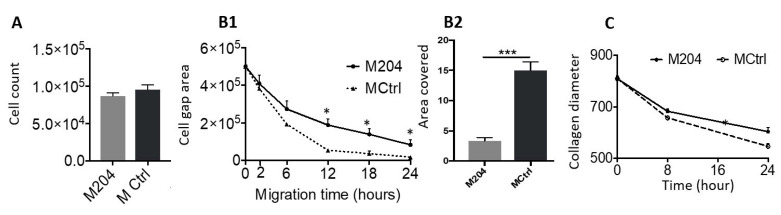
Regulation of (**A**) CAF proliferation, (**B1**) CAF migration alone, and (**B2**) in interaction with UK1 and (**C**) CAF collagen contraction ability post miR-204 transfection (*n* = 6) (* *p* < 0.05, *** *p* < 0.0005).

Proliferation of CAFs was not altered on increasing expression of miR-204 using miR mimics (Figure 5A). Migration and collagen contraction are essential attributes of CAFs, proven to be essential for adjacent OSCC cell invasion [20]. Increased expression of miR-204 resulted in decreased migration of CAFs alone (Figure 5B1) or in interaction with OSCC cell line UK1 (Figure 5B2) in 2D monolayer migration assay. Increased expression of miR-204 in the fibroblasts resulted in decreased collagen I gel contraction (Figure 5C).

### 2.6. Expression of miR-204 in Fibroblasts Inhibits Invasion and Migration of Adjacent OSCC Cells

**Figure 6:**
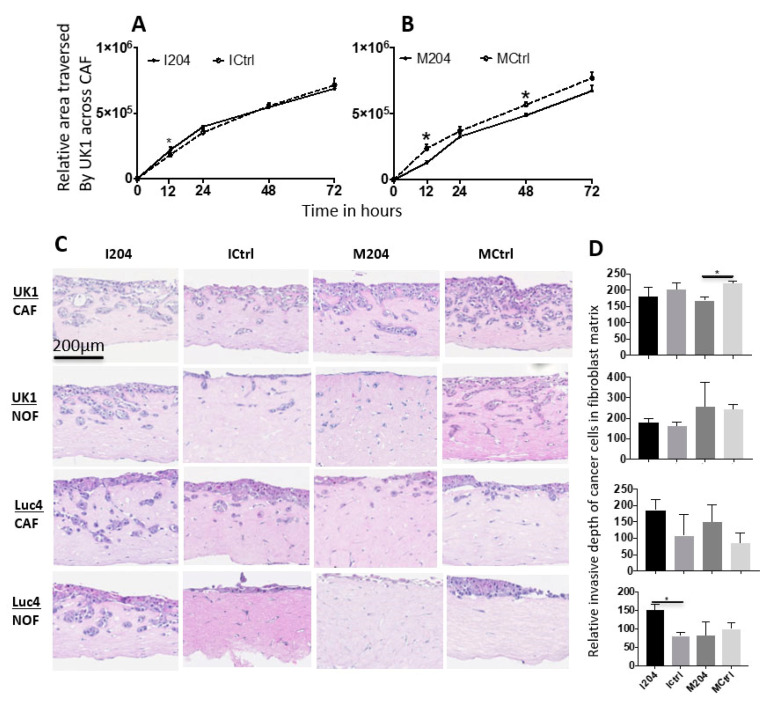
(**A**,**B**) Graphs showing quantification of the area traversed by UK1 across the gap towards CAFs at different time points from the baseline post miR-204 modulation in CAFs in UK1-CAF monolayer co-culture (*n* = 4). (**C**) Representative pictures from 3D organotypic cultures showing the invasion by OSCC cell lines UK1 and Luc4 in post miR-204 modulated fibroblasts-populated collagen matrices and (**D**) the corresponding graphs depicting the quantification of invasion depths (*n* = 4, unpaired *t*-test, * *p* < 0.05).

When co-cultured in 2D monolayers with CAFs treated with miR-204 inhibitors, only minimal effects on migration of OSCC cell line UK1 were observed (Figure 6A). When UK1 were co-cultured with CAFs treated with miR-204 mimics, UK1 cells showed a significant reduction of migration across the gap area towards the CAF region (Figure 6B). In 3D organotypic co-culture models, increased expression of miR-204 in CAFs significantly reduced the depth of invasion by UK1 cells, while inhibitors of miR-204 in NOFs significantly increased the depth of invasion of OSCC cell line Luc4 (Figure 6C,D).

### 2.7. miR-204 in Fibroblasts Regulates Several Molecules Involved in Cell Migration

**Figure 7:**
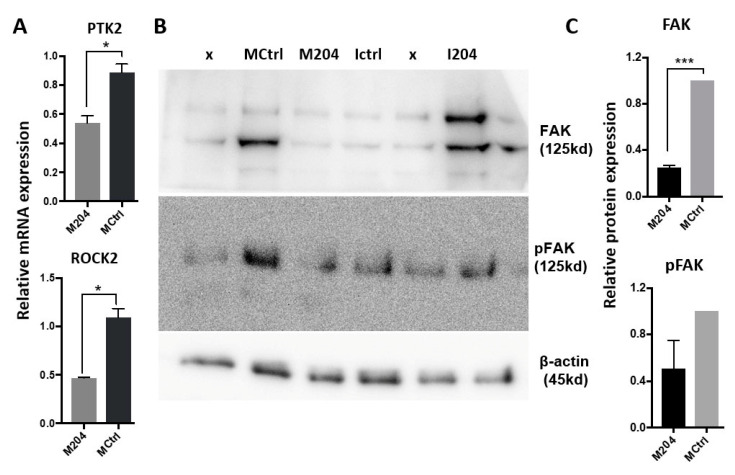
Changes in (**A**) mRNA and (**B**) protein expression following miR-204 modulation in CAFs. Western blot image (**B**) and the semi-quantification of protein blots using ImageJ (**C**) (* *p* < 0.05, *** *p* < 0.0005).

Increased miR-204 expression in CAFs significantly decreased the levels of PTK2 (FAK) and ROCK2 transcripts (Figure 7A). Moreover, at the protein level, FAK and its active form, phosphorylated FAK (pFAK), were decreased upon increased miR-204 (Figure 7B,C).

## 3. Discussion

In this study several miRs were identified to have a differential expression in CAFs compared with NOFs, as demonstrated by the segregation of CAF and NOF strains in two separate clusters by unsupervised clustering analysis of the miRs expression in the miR array. Nevertheless, one of the NOF strains exhibited a miR expression profile similar to CAF strains, and two of the CAF strains displayed a miR expression profile similar to that of NOF strains. We have previously demonstrated CAF heterogeneity at transcriptomic level by gene microarray analysis [19]. The present study points towards CAF heterogeneity regarding miR expression as well. One possible explanation for this variation could be the fact that the fibroblasts used for miR array in this study were primary cells in their early passages, and that these differences most likely reflect the patient-to-patient biological variation and heterogeneity. Nevertheless, there could be other reasons for this heterogeneity such as selection in culture. While miR-204 expression was increased in almost all cases of NOFs isolated from healthy, non-cancer patients compared with the CAFs isolated from OSCC lesions, miR-204 expression was much lower in two of the CAF–NOF pairs, in which NOFs were isolated from the morphologically normal mucosa of OSCC patients. While these differences might point to differences in the biology of miR regulation between NOFs from non-cancer-related and cancer-bearing individuals, the possible alteration of NOFs in OSCC cancer patients cannot be excluded, taking into consideration the field cancerization phenomenon common for the oral mucosa of OSCC patients, in which pre-malignant or even malignant alterations in the apparently normal tissue surrounding the tumor are quite common [38]. In this line, a study by Ganci F.; et al. showed that miR regulation was different from tumor to peri-tumor region and from peri-tumor to normal surrounding in HNSCC [39]. The tumor cells and tumor microenvironment (TME) co-evolve [26], meaning that transformations in all TME components including fibroblasts take place alongside neoplastic transformation of the epithelium from early stages. Increased heterogeneity of fibroblasts when seeded on plastic as 2D monolayers might as well be related to the selection of certain populations that survive in culture. One then might question the representability of the fibroblasts isolated from the tissues in cell culture for the fibroblast population in that tumor or tissue. Changes in cells’ phenotype upon transitioning from tissue to cell culture have also been reported [40], thus use of culture conditions as close as possible to the in vivo situation, such as 3D collagen gels or 3D organotypic co-cultures, is essential.

The major limitation of our study is the use of a limited number of fibroblast strains, although comparable with previously published studies on miRNA array in CAFs versus NOFs. Few other miRNA array studies on pairs of CAFs versus normal fibroblasts (NF) have been previously performed [41,42,43,44]. Significantly dysregulated miRNA candidates in CAF in our OSCC study were distinct from those miRNA identified by the previous studies, and this could be attributed to different localization of the cancers from which fibroblasts were isolated. The eight miRNA found significantly downregulated in CAFs in our study, among which was also miR-204, affected TGFβ signaling, adherens junction, and proteoglycans in cancer pathways among other pathways significantly affected by them, as shown by KEGG pathway analysis by Diana MirPath v.3 using a database of experimentally supported miRNA–gene interactions: TarBase v.7 and [45]. Among the pathways significantly affected by the four miRNAs found to be upregulated in CAFs versus NOFs in our study were p53 signaling, cell cycle, and proteoglycans in cancer pathways.

For validation on different data sets, we first explored GEO datasets on miR-expression in CAFs versus NOFs in different cancers. We identified three studies that had compared miRNA expression in CAFs versus NOFs. With GEO2R analysis, we did not match any significantly deregulated miRNA candidates identified in our OSCC study with those in prostate CAFs (*n* = 3) versus NOFs (*n* = 3) (GSE68166; GPL10558). Another dataset (GSE97545) in lung cancer could not be analyzed. The data from the third published study on miRNA array in CAFs versus NOFs in OSCC were not made publicly available, so we could not investigate that dataset either [43]. Therefore, we sought next to explore the expression of the miR, thus we further focused our attention in this study on miR-204, in OSCC versus normal oral mucosa in the TCGA database. In spite of the fact that TCGA miRNA data were derived from the whole OSCC tissues, a decrease in miR-204 expression in OSCC compared with normal tissues (statistically significant for matched, paired lesions) was observed, which is in line with the decreased miR-204 in CAFs compared with NOFs observed in our study.

Among the twelve dysregulated miRNA identified in our study, we focused further on miR-204 in functional and molecular assays owing to a possible link that we found in silico between this particular miRNA and integrin alpha11, which has been identified as one of the top genes significantly up-regulated in CAFs derived from OSCC in our previous transcriptomic study [19]. Supportive of our choice was also the fact that earlier studies have suggested tumor-suppressive functions by miR-204, as described in the Introduction. Reduced expression of miR-204 in cancers, likening or leading to aggressiveness and metastasis of cancer cells, was previously reported. We have previously investigated miR204 expression and its correlation with clinical-pathological parameters and survival in a cohort of 169 HPV-negative OSCC patient cohort [36]. Data from that cohort showed a correlation between the expression of miR-204 in tumor center and the degree of differentiation, namely higher miR-204 expression in well-differentiated OSCC lesions. However, we could not find any correlation between the differentiation degree of the OSCC lesions from which the CAFs were derived and the expression of miR204 in the isolated CAFs, probably owing to the heterogeneity of the CAFs within a tumor or because of the low number of cases in which we isolated CAFs for analysis in the present study.

However, it was previously unknown how miR regulation in CAFs in general, and miR-204 expression in particular, can modulate the tumor promoting function by CAFs. In order to identify the role of miR-204 in CAFs, CAFs and respective matched NOFs from two OSCC patients were miR-204 modulated and used to populate the collagen matrix of a 3D organotypic model in which OSCC cells could be tested for their invasive potential. Increasing miR-204 expression in CAFs suppressed OSCC cell invasion. On the contrary, when miR-204 expression was inhibited in paired NOFs, invasion of OSCC cells increased significantly. These findings, coupled with the decreased collagen contraction and migration of CAFs when treated with miR204 mimics, indicate a tumor suppressive role of miR-204 expression in CAFs in OSCC by affecting fibroblast motility [20].

Our previous work on transcriptomic analysis of the same CAF and NOF strains showed upregulation of ITGA11 in CAF derived from OSCC lesions [19]. Moreover, we described a correlation with a CAF phenotype for CAFs expressing high levels of ITGA11 [24] and a tumor-promoting role of ITGA11 when expressed in CAFs [46]; integrin α11 is a collagen I receptor, and it has been involved in cell motility [47]. In this study, integrin α11 expression was increased in CAF compared with NOF strains in all five matched pairs, confirming the previous results on non-matched CAFs and NOFs [19]. miR target prediction analysis showed that ITGA11 contains a target site for miR-204. Therefore, we performed luciferase miR target reporter assay and demonstrated that, indeed, ITGA11 is directly targeted by miR-204, affecting ITGA11 expression at both transcript and protein levels. Together with our data on miR-204 affecting fibroblast cell motility, this suggests that the tumor suppressive effect of miR-204 is mediated via ITGA11. Nevertheless, miR-204 is only one of the several miRs that regulate ITGA11. In addition, miR-204 has also been documented to affect other molecules and pathways of cancer. In this line, we show in this study that several other CAF-related molecules were modulated by miR-204, endorsing also a more complex role of miR-204 in regulating the CAF phenotype than only through ITGA11.

Our molecular analysis targeted towards motility-related molecules showed that modulation of miR-204 expression significantly altered, in addition to ITGA11, the mRNA levels of FAK and ROCK2, two other molecules playing an important role in cell motility and CAF motile phenotype. FAK has been shown to be involved in actin filaments-based protrusions, named filopodia via polymerization and bundling of linear actin filaments within fan like lamellipodia, and thereby affecting cell adhesion and motility [48]. FAK may be activated by integrins, receptor tyrosine kinases (RTKs), mechanical stimuli, cytokine and G-protein coupled receptors (GPCRs), and changes of intracellular pH (H+) [48]. This study adds one more level of complexity in the regulation of FAK, showing that, in addition to post-translational modifications, expression of FAK transcripts and total FAK protein levels are regulated by miR-204. ROCK2 is another oncoprotein that controls cytoskeleton organization and cell motility. ROCK2 overexpression has been reported in OSCC-CAFs and it has been shown to have a prognostic value for OSCC and to be associated with CAF density [49]. Our current findings that miR-204 targets several motility-related molecules corroborate well with our functional results showing that miR-204 alters the motility of fibroblasts, and are in line with previous observations of miR-204 affecting cell motility, although this has been shown previously in cancer cells only [50]. Furthermore, the alterations in CAF motility observed when miR-204 mimics were used were followed by impaired OSCC cell invasion, in line with the previous studies pointing to the essential role of CAF motility for fibroblast-led collective OSCC cell invasion [20]. This is an intricate mechanism suggesting a tumor-suppressive role for miR-204 though regulation of CAF motility via restriction of several motility-related molecules and direct regulation of the collagen receptor integrin α11.

## 4. Materials and Methods

### 4.1. Patient Material

CAFs were isolated from biopsies of OSCC primary lesions of patients (*n* = 13) diagnosed at Haukeland University Hospital, Bergen, Norway, prior to any treatment. Matched normal oral fibroblasts (NOFs) were also isolated from biopsies taken from morphologically cancer-free regions of oral mucosa of five of the OSCC patients (*n* = 5) (Supplementary Table S1). Tissues from the patients who underwent neoadjuvant therapy and/or later were confirmed HPV-positive were not included in the study. NOFs from biopsies of normal oral mucosa of healthy, cancer-free volunteers (*n* = 9) were also obtained. Outliers, one each from the CAF and NOF groups, were removed in miR-microarray data analysis. Written consent was obtained from both OSCC patients and healthy donors. Tissues were collected in Dulbecco’s modified Eagle’s medium (DMEM; D6429, Sigma), supplemented with 2% antibiotic-antimycotic (AB/AM; 100 U/mL penicillin, 100 ug/mL Streptomycin, and 25 ng/mL Amphotericin B; all from Invitrogen, Waltham, MA, USA). Ethical approval was obtained from the regional ethical committee in Norway (West Norway; REKVest 3.2006.2620, REKVest 3.2006.1341).

### 4.2. Fibroblast Isolation and Cell Culture

Tissues collected were thoroughly washed in the collecting medium. Bleeding and necrotic areas of the tissues were removed using a sterile scalpel and washed thoroughly again. Tissues were cut into approximately 2–4 mm^3^ bits, and then placed on 6 cm culture dish. Tissue bits (explants) were slightly air-dried (approximately 1–2 min) to facilitate explants to attach on the surface of the dish. Thereafter, explants were carefully covered with complete growth medium, DMEM with 10% heat inactivated newborn calf serum (NBCS; 31765068, Gibco, Amarillo, TX, USA) supplemented with 1% AB/AM. The culture dishes were then incubated in a growth chamber maintained at 37 °C and 5% CO_2_. Few days later, cells with epithelial morphology that grew from the explant were scrapped off under a microscope using pipette tips. Alternatively, cells with fibroblast morphology were trypsin selected (trypsin detached fibroblasts earlier compared with epithelial cells; 1–2 min vs. 4–7 min). CAFs and NOFs obtained were further propagated in complete DMEM medium without AB/AM. All functional assays were performed in matched fibroblast pair 21.

OSCC cell lines UK1 [51] and Luc4 [52] were grown in DMEM/Nutrient Mixture F-12 Ham (DMEM/F12; D8437, Sigma, St. Gallen, Switzerland), supplemented with 10% NBCS, 0.4 µg/mL hydrocortisone (H0888, Sigma), 1× Insulin–Transferrin–Selenium (41400-04, Thermofisher Scientific), 50 µg/mL L-ascorbic acid (A7631, Sigma), and 10 ng/mL epidermal growth factor (E9644, Sigma).

### 4.3. Total RNA Isolation and Small RNA Enrichment for miR Microarray

RNA was isolated using miRVana RNA extraction kit (AM1561, Thermo Fisher Scientific, Dreieich, Germany) following the manufacturer’s protocol. In brief, sub-confluent cell cultures were washed with tepid phosphate buffered saline (PBS), lysed with lysis/binding buffer and phenol/chloroform was extracted. For total RNA isolation, the aqueous phase with RNA from phenol/chloroform extraction was treated with 1.25 volume of 100% ethanol. RNA was then captured in glass fiber filter cartridge and collected in 100 μL of preheated (95 °C) nuclease free elution solution (0.1 mM EDTA).

For the enrichment of small RNAs (~200 nucleotides and less) for miR microarray, 50 μg of total RNA was mixed with five volumes of lysis/binding buffer. To the RNA mixture was added 1/10 volume of homogenate additive, after which it was mixed well and left on ice for 10 min. The RNA mixture was then mixed thoroughly with 1/3 volume of 100% ethanol to allow capture of larger RNA in the filter cartridge. The filtrate with small RNAs was collected, 2/3 volume of 100% ethanol was added, and it was passed through a new filter cartridge. The cartridge was washed with Wash Solutions and, finally, small RNA was collected in elution solution.

The purity and quantity of the isolated RNAs were measured using NanoDrop^®^ ND-1000 Spectrophotometer and ND-1000 V3.5.2 software (Nanodrop Technologies, Wilmington, DE, USA). RNA was quantified using the principle that an absorbance reading of 1 at 260 nm wavelength is equivalent to 40 μg/mL of RNA. The blank measurement was made with 1 μL of elution buffer; 1 μL of the RNA samples were pipetted on the measurement pedestal and the absorbance was taken at 260 nm, 280 nm, and 230 nm wavelength. Purity was assessed assuming that the pure RNA has an A260/A280 of 2.1 and A260/A230 of 2. Isolated RNAs were stored at −80 °C.

### 4.4. miR Microarray

CAFs and NOFs at early passages (*p* < 5) were grown in 3D in collagen gels (rat tail, BD Biosciences, Franklin Lakes, NJ, USA) containing DMEM, NBCS, and reconstitution buffer (2.2g NaHCO_3_ + 0.6 g NaOH + 4.766 mL HEPES in 100 mL double distilled water H_2_O) at a volume ratio of 7:1:1:1, respectively, at the concentration of 4 × 105 cells/mL. After 5 days in 3D culture, cells were harvested. Then, 100 ng of enriched small RNAs isolated from the cells was converted cDNA and fed into Illumina microarray version 2 panel with miR-specific oligos. The samples were profiled for 1146 human miRs expression (>97% coverage from miRBase release 12). Using J-Express [31,53], the data file from miR microarray was normalized, outliers; one each from CAFs and NOFs were removed; and, with significant analysis of microarray (SAM), significantly differentially expressed miRs in between CAFs and NOFs were identified. The differentially regulated miRs were further validated by miR specific reverse transcription (Applied Biosystems, Waltham, MS, USA) and quantitative real time PCR (qRT-PCR) using Taqman assays (Supplementary Table S2).

### 4.5. Reverse Transcription

For miR quantification, miRs specific primers were used to synthesize specific miR cDNAs (TaqMan MicroRNA Reverse Transcriptase kit, Applied Biosystems). In brief, 10 ng of total RNA was mixed with miR specific primer, dNTPs, reverse transcription buffer, RNAse inhibitor, and reverse transcriptase to the final reaction mixture volume of 15 µL. The reaction mixture was then subjected to a thermal cycle of 16 °C for 30 min, 42 °C for 30 min, and 85 °C for 5 min in Mastercycler Gradient thermal cycler (Eppendorf).

For mRNA quantification, reverse transcription of total RNA into cDNA was carried using Taqman Reverse Transcription kit (Applied Biosystems). In brief, 100 ng of RNA was mixed with reverse transcription buffer, random hexamers, MgCl2, dNTPs, RNase inhibitor, and reverse transcriptase and adjusted to a final volume of 25 μL with RNase free water. The reaction mixture was subjected heated at 20 °C for 10 min, 48 °C for 30 min, and 90 °C for 5 min.

### 4.6. Quantitative Real-Time Polymerase Chain Reaction (qRT-PCR)

miRs and mRNAs were quantified by qRT-PCR using Taqman Gene Expression assays in ABI Prism 7900 HT sequence detector system (Applied Biosystems). In 384-well reaction plates (Thermo Fisher Scientific), PCR reaction volume of 10 μL in each well was prepared by mixing 1 μL of cDNA with 1 μL Taqman assay, 5 μL Universal Master Mix, and 3 μL RNAse free water. The plate was then run at 50 °C for 2 min, 95 °C for 10 min, and 40 cycles of 95 °C for 15 s and 60 °C for 1 min. Each sample was run in triplicate. A threshold cycle of mRNA obtained was normalized to endogenous controls GAPDH. GAPDH was further verified with 18S and RPL13A. miR expression was normalized to RNU48. Relative expressions were calculated using the 2^−ΔΔCt^ method.

### 4.7. TCGA Data Analysis

TCGA data on miR-204 expression in head and neck squamous cell carcinoma (HNSCC) and normal oral tissues and the associated clinical data were obtained from Firebrowse database version 2016_01_28 (http://www.firebrowse.org; accessed on 30 April 2020). HPV-positive cases, non-oral cancer cases, and cases with history of neoadjuvant therapy were excluded from the cohort. Therefore 251 OSCC and 21 normal oral cases remained after exclusion. Of 21 cases with normal mucosa, data for 20 matched OSCC cases were available.

### 4.8. miR Target Identification and miR Dual Luciferase Target Reporter Assay

miR target prediction by miR target prediction softwares TargetScan Release 7:1 [54], miRmap [55] and miRDB [56] showed target conserved sites for several miRs in the 3′ UTR of ITGA11. Of the three, TargetScan and miRmap predicted target site for miR-204 in the 3′ UTR of ITGA11 (3′ UTR ITGA11 sequence: 5′ GGCTCCAGAGGAGACTTTGAGTTGATGGGGGCCAGGACACCAGTCCAGGTAGTGTTGAGACCCAGGCCTGTGGCCCCACCGAGCTGGAGCGGAGAGGAAGCCAGCTGGCTTTGCACTTGACCTCATCTCCCGAGCAATGGCGCCTGCTCCCTCCAGAATGGAACTCAAGCTGGTTTTAAGTGGAACTGCCCTACTGGGAGACTGGGACACCTTTAACACAGACCCCTAGGGATTTAAAGGGACACCCCTAACACACCCAGGCCCATGCCAAGGCCTCCCTCAGGCTCTGTG 3′). 3′UTR sequence of ITGA11 (NM 001004439.2) with miR-204 binding site was retrieved from UCSC genome browser (http://genome.ucsc.edu, accessed on 17 November 2019) [57], and a plasmid vector with luciferase upstream of 3′UTR and renilla as a transfection control under different promoters were designed using VectorBuilder. Non-complimentary mutant sequence was introduced to the miR-204 binding site (236–242 AAAGGGA → ATTCCCT) on 3′UTR of ITGA11 as control. Both vectors were purchased from VectorBuilder. Reduction in luciferase signal normalized to renilla signal compared with mutant control after miR-204 mimics transfection into the cells indicates direct targeting of ITGA11 by miR-204. Luciferase and renilla activity was measured using Dual-Luciferase Reporter Assay (Promega; E1910) following the manufacturer’s protocol using a luminometer. In brief, 5 × 10^4^ CAFs in each well in 24-well plates were reverse co-transfected with 250 ng of plasmid DNA and miR-204 mimic at a 50 nM concentration in complete DMEM medium using LipofectamineTM 3000 Transfection Reagent. Then, 48 h after transfection, growth media from cultured cells were removed and washed with PBS solution. Thereafter, the cells were lysed with 100 µL of 1X lysis buffer. The cell culture plate was gently rocked for about 15 min to allow cells to lyse completely. Then, 20 µL of cell lysates were pipetted into the wells in 96-well plates and dispensed with 100 µL of luciferase substrate. Immediately, luciferase activity was read using a Tecan Infinite M200PRO luminometer. The wells were again dispensed with 100 µL of 1X Stop & Glo reagent and renilla luciferase activity was read.

### 4.9. miR-204 Modulation in Cultured Fibroblasts

Fibroblasts were reverse transfected using LipofectamineTM 3000 Transfection Reagent (L3000015; Invitrogen) with a mimic (C-300563-05, Dharmacon, Lafayette, CO, USA), an inhibitor (IH-300563-07, Dharmacon), and the respective controls (CN-001000-01, IN00105-01-05, Dharmacon) of miR-204-5p at 50 nM concentration in the growth medium. Cells were harvested for RNA or protein extraction or subjected to further experiments after 48 h of transfection. Transfection of miR-204 mimic induced a significant increase in mir-204 levels in both CAF and NOF strains (Supplementary Figure S1), while transfection of miR-204 inhibitor did not induce a significant change in the miR-204 levels in cultured fibroblasts (Supplementary Figure S2).

### 4.10. Protein Isolation and Quantification

The cell cultures were washed twice with ice cold PBS buffer, lysed with ice cold RIPA lysis and extraction buffer (89901, Thermo Fisher Scientific), supplemented with 1× Halt Protease and Phophatase Inhibitor (78442, Thermo Fisher Scientific), and scrapped using sterile scrapers. The lysates were then collected in ice-cold Eppendorf tubes and centrifuged at 14,000 rpm for 15 min. Protein supernatants were aliquoted in new Eppendorf tubes and stored at −80 °C until use.

Total protein in the lysates was measured by DC protein assay (5000111, BioRad, Hercules, CA, USA) in a 96-well microplate, using the manufacturer’s protocol. In brief, 20 μL of reagent S was mixed with 1 mL of reagent A to give a working reagent A′. Then, 1.5 μg/mL of BSA was diluted in twofold to use as standards. Five microlitres of protein samples and standards were then pipetted into a microtiter plate, and 25 μL of reagent A′ and 200 μL of reagent B were added. The plate was gently agitated to mix the reagents, incubated at room temperature for 15 min, and the optical density was measured at 750 nm wavelength using (BIO-TEKR). All the samples were measured in triplicates, and blanks were included.

### 4.11. Western Blotting

Fifteen micrograms of protein were mixed with 4× NuPAGE LDS sample buffer (NP0007, Invitrogen) and 10× NuPAGE sample reducing agent (NP0004, Invitrogen) to the final concentration of 1×. The protein mixture was heated at 95 °C for 5 min and run on NuPAGE Novex 10% Bis-Tris Protein Gel (NP0303, Invitrogen) in 1× NuPAGE MOPS SDS Running Buffer (NP0001, Invitrogen) at 160 Volt for 90 min. Then, 500 μL of NuPAGE Antioxidant (NP005) was added in the upper chamber immediately before applying the voltage. Precision Plus Protein Dual Color Standard (1610374, Biorad) was used as a protein weight marker. The resolved protein in the gel was then blotted into methanol activated (for 1 min) Amersham Hybond P 0,45 PVDF membrane (10600069, GE Healthcare, Chicago, IL, USA) using filter paper sandwich in 1x NuPAGE transfer buffer (NP0006, Invitrogen) with 10% methanol and 0.2% Antioxidant at 40 Volt for 1 h. Then, 200 μL of antioxidant was added in the upper chamber immediately before applying the voltage.

After completion of the blotting, PVDF membrane was blocked with 5% dry milk or 3% BSA prepared in 1× Pierce TBS Tween 20 Buffer for 30 min. The PVDF membrane was then incubated with primary antibody in 5% non-fat dry milk or 3% BSA for overnight at 4 °C. Thereafter, the membrane blot was washed with TBS-Tween three times for 10 min each and incubated with secondary antibody for one hour at room temperature (anti-rabbit from Cell Signalling, #7074, and anti-mouse from Cell Signalling, #7076). The membrane was then washed with TBS-Tween four times for 10 min each and, finally, visualized using SuperSignal West Pico Chemilluminescent Substrate (34080, Thermofisher) in Image Reader LAS 1000 (Fujifilm). Relative protein amounts in each protein bands in the captured images were quantified using ImageJ using Gel commands. Antibodies used for Western blotting are listed in Supplementary Table S3.

### 4.12. Migration Assay (2D Co-Culture)

Two-well silicone inserts (80209, ibidi) were placed in the center of 24-well plates. Fibroblasts or OSCC cells (2 × 10^4^ cells) were plated, and reverse transfected with miR mimics and inhibitors) into each well. Silicone inserts were removed 48 h post-transfection to create a uniform cell-free gap of 50 µm between the edges of two cell-rich zones. The wells were then imaged at two-hour time interval using IncuCyte Zoom using a 4× objective (EssenBioScience, Ann Arbor, MI, USA). The cell-free unoccupied area was measured using MRI Wound Healing Tool in ImageJ.

### 4.13. Collagen Contraction Assay

Ninety-six-well plates were blocked overnight with 2% BSA in PBS in 37 °C incubation chamber. Forty-eight hours post-miR modulations, fibroblasts at 5 × 10^5^/mL density were uniformly suspended in collagen type I matrix, as described above. Subsequently, 100 µL of collagen cell suspension was added into each well and allowed to gel for 90 min. The gels were then floated with 100 µL of DMEM medium, and the change in gel dimensions was measured at different time points.

### 4.14. 3D Organotypic Co-Cultures

Fibroblasts at a density of 5 × 10^5^ cells/mL were re-suspended in collagen type I (rat tail, 354239, BD Biosciences, Franklin Lakes, NJ, USA) matrix, and uniformly mixed with DMEM, NBCS, and sterile reconstitution buffer in the volume ratio of 7:1:1:1, respectively. Then, 700 µL of the collagen cell suspension was dispensed into each well in 24-well plates and allowed to gel in a humidified incubator at 37 °C. After two hours, the gels were added to 1 mL of fibroblast growth medium to allow the cells to grow until next day. The next day, 5 × 10^5^ OSCC cells were added on the top of each fibroblast gel. Another day, the gels were transferred on the metal grids layered with a filter paper. Organotypic growth medium (DMEM and Ham’s F12 Nutrient mixture (31765068, Thermo Fisher Scientific) in ratio 1:3, and supplemented with Insulin-Transferrin-Selenium, hydrocortisone, and L-ascorbic acid, as mentioned above, and NBCS replaced with 0,1% Bovine Albumin Fraction (V15260-037, Thermo Fisher Scientific)) was added to reach the level of filter paper to allow gels to grow at the air–medium interface. Medium was changed at each alternative day. Ten days after culture on grid, gels were fixed overnight in 4% buffered formalin and embedded in paraffin.

### 4.15. Quantification of Invasion of OSCC Cells in 3D-Organotypic Models

Tissue gels embedded in paraffin blocks were cut into 5 μm sections and stained with hematoxylin and eosin. Invasion depth of OSCC cells in the stained organotypic sections was measured using NDP.view2 (Hamamatsu, Naka Ward, Sunayamacho, Japan). Invasion depth was measured as the vertical distance of invaded cancer cells from the reconstructed basement membrane (horizontal line along the non-invading cells). Twenty measurements at each 50 µm distance along the tissue were taken and averaged. Non-uniform thick, curved, or tapered ends of the 3D organotypic tissues as a result of differential contraction by the fibroblasts were excluded in the analysis.

### 4.16. Statistical Analysis

SAM analysis was performed to detect differentially regulated miRNAs using J-Expres (University of Bergen, Norway), a freely available software for analyzing microarray gene expression data [58]. Differentially expressed miRs with false discovery rate (FDR) = 0 were considered to be significantly modulated miRs. For functional assays, and miR, mRNA, or protein expressions data, paired and unpaired *t*-tests were applied to find the significant difference in means (*p* < 0.05). For non-normally distributed data (D’Augostino & Pearson test; *p* > 0.05), non-parametric comparisons (Mann–Whitney for unpaired comparison between two, and Kruskal–Wallis for unpaired comparison among groups) were carried out to find significant differences in median expression. Parametric and non-parametric analyses were carried out as required using GraphPad Prism 7 (GraphPad, San Diego, CA, USA).

## 5. Conclusions

This study demonstrates for the first time miR dysregulation in OSCC-derived CAFs compared with NOFs and identifies twelve differentially expressed miR in CAFs isolated from OSCC lesions compared with fibroblasts isolated from normal mucosa. One of the dysregulated miRs, miR-204, was further investigated, and we show here for the first time that miR-204 directly targets the ITGA11, and that its tumor-suppressive function is mediated via alteration of CAF motility though regulating several other motility-related molecules in addition to ITGA11.

## Data Availability

The datasets for this study are available here. GEO accession GSE172287: Go to https://www.ncbi.nlm.nih.gov/geo/query/acc.cgi?acc=GSE172287.

## Supplementary Materials

The following are available online at https://www.mdpi.com/article/10.3390/ijms222111960/s1.
