# Supplementary material for: Profiling and Functional Analysis of microRNA Deregulation in Cancer-Associated Fibroblasts in Oral Squamous Cell Carcinoma Depicts an Anti-Invasive Role of microRNA-204 via Regulation of Their Motility
Source: Int J Mol Sci. 2021 Nov 4;22(21):11960. doi: 10.3390/ijms222111960 (PMC8584862; doi:10.3390/ijms222111960)
Supplement: Supplementary file 1 [file ijms-22-11960-s001.zip › ijms-1425326-supplementary/supplementary tables R1.pdf]

Supplementary Table S1. Patient information

ID	*Age Gender*	*site*	*TNM*	*Differentiation*	*Later metastasis*	*Smoking*	*Survival (months)*
Donors of CAF-NOF matched pairs							
7m	68, M	gingiva	T4N2M0	middle	bone, lung	yes	20
8m	69, M	floor of mouth	T4N1M0	middle	bone	yes	9
10m	51, M	tongue	T2N2bM0	high	no	-	alive
15m	66, M	gingiva	T4N0M0	poor	-	yes	1
21m	62, M	tongue	T2N1M0	high	lung, liver	yes	8
Donors of CAF (un-matched group)							
7	80, F	buccal	T2N2M0	high	no	no	12
9	66, F	buccal	T2N2M0	high	no	no	alive
10	49, M	tongue	T3N1M0	middle	no	yes	96
11	57, M	tongue	T4N2M0	middle	-	-	-
12	52, M	tongue	T3N1M0	poor	-	-	-
13	72, M	floor of mouth	T4N1M0	middle	-	yes	-
24	63, F	tongue	T3N1M0	high	bone	no	16
25	42, M	gingiva	T4N1M0	high	lung, bone	no	12

Supplementary Table S2. List of Taqman Assays (ThermoFisher, USA)

Genes	*Taqman Assay ID*
hsa-miR-204-5p	000508
hsa-miR-138-5p	002284
hsa-miR-582-5p	001983
RNU48	001006
ITGA11	Hs00201927_m1
PTK2	Hs01056457_m1
EGFR	Hs01076090_m1
GAPDH	Hs99999905_m1
FAP	Hs00990806_m1
TGFB1	Hs00998130_m1
ROCK2	Hs00178154_m1
18s	Hs99999901_s1
TGFBR2	Hs00559660_m1
RPL13A	Hs04194366_g1

Supplementary Table S3. List of antibodies used for western blot.

1o Antibodies	*Cat No*	*Dilution*	*Protein block*
ITGA11 [1]	-	1:2000	5% Dry milk
FAK (D2R2E)	#13009, Cell Signalling	1:1000	3%BSA
p-FAK(Tyr397) (D20B1)	#8556, Cell Signalling	1:1000	3%BSA
FAP (SP325	#ab207178 Abcam	1:1000	5% Dry milk
TGFBR2 (E5M6F)	# 41896S, Cell Signalling	1:1000	5% Dry milk
GAPDH	AB9484, Abcam	1:10000	5% Dry milk
B-Actin	#4967, Cell Signalling	1:5000	5% Dry milk

1. Velling T, Kusche-Gullberg M, Sejersen T, Gullberg D: **cDNA cloning and chromosomal localization of human alpha(11) integrin. A collagen-binding, I domain-containing, beta(1)-associated integrin alpha-chain present in muscle tissues**. *J Biol Chem* 1999, **274**(36):25735-25742.
