# Supplementary figures and images for: Profiling and Functional Analysis of microRNA Deregulation in Cancer-Associated Fibroblasts in Oral Squamous Cell Carcinoma Depicts an Anti-Invasive Role of microRNA-204 via Regulation of Their Motility
Source: Int J Mol Sci. 2021 Nov 4;22(21):11960. doi: 10.3390/ijms222111960 (PMC8584862; doi:10.3390/ijms222111960)

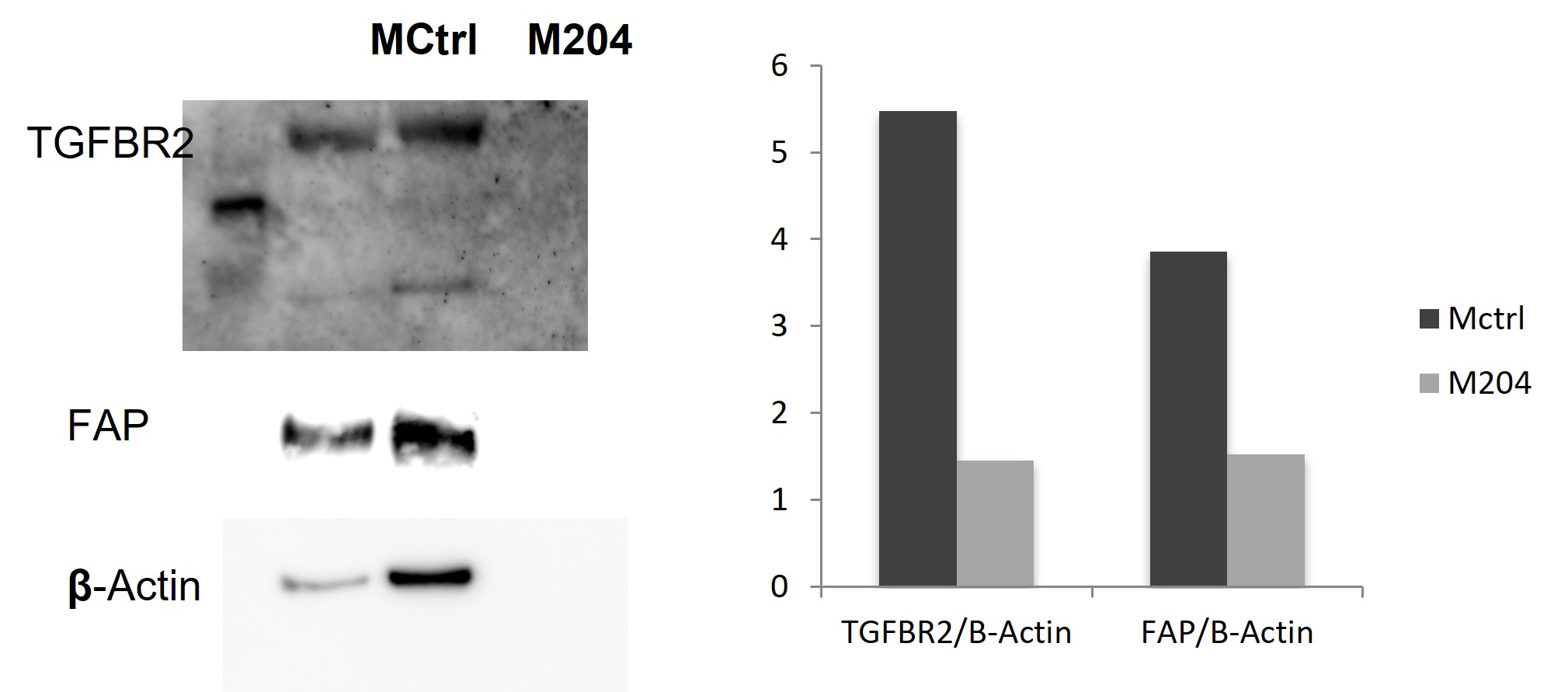

Supplement: Supplementary file 1 [file ijms-22-11960-s001.zip › ijms-1425326-supplementary/Fig S1 IJMS.jpg]

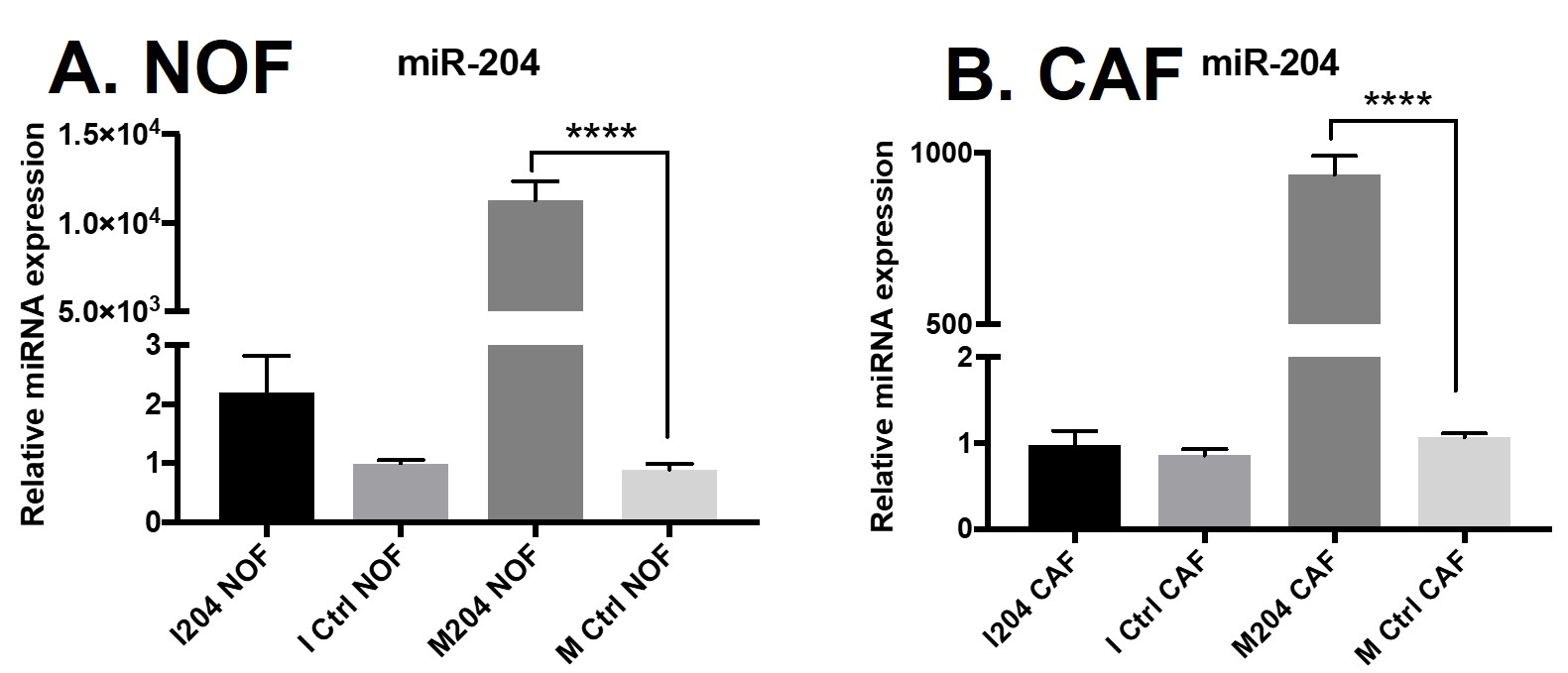

Supplement: Supplementary file 1 [file ijms-22-11960-s001.zip › ijms-1425326-supplementary/Fig S2 IJMS.jpg]
